# Chemical Affinity

**Published:** 1873-10

**Authors:** D. Vaughan


### CHEMICAL AFFINITY.

BY D. VAUGHAN.

Nothing contributes more to the advancement of physical science, than the means occasionally devised for attaining accuracy and precision, in theoretical and experimental research. The establishment of the doctrine of definite proportions, has been of incalculable advantage to the progress of chemistry; but while in weight and measure, combining elements conform to the strict accuracy of numerical relations, no such conformity has been hitherto traced in the forces which produce such combinations. During the last century, Bergman supposed that the affinity between acids and bases was invariable, that it could be numerically estimated, and that the causes of decomposition could be always traced by very simple laws. But Berthollet pointed out the fallacy of Bergman's doctrine and showed that the course of chemical changes were not immutable, that they could be often reversed, and that they were dependent on certain influences apart from the mutual attraction between the combined bodies.

According to Berthollet, when salts are in solution at common temperature, the occurrence of a decomposition mainly depends on the insolubility of the resulting compounds. Thus, on mixing together in solution nitrate of lime and carbonate of potash, there will be formed nitrate of potash and carbonate of lime ; the latter being precipitated in an insoluble form. This insolubility, or the cohesive force on which it was supposed to depend, Berthollet regarded as the controlling cause of the decomposition. In cases where no insoluble compounds are formed, he supposed that the principle of cohesion would lead to the formation of such salts as were least soluble and were first precipitated from the solution. In a dry state and at a high temperature, he supposed that the chemical changes were determined by the volatility of the resulting compound, and in this way he accounted for the fact that in these circumstances the course of decomposition is often found to be diametrically opposite to that observed in solution.

Berthollet was far less reasonable in supposing that an excessive quantity of an acid or a base would compensate for its weakness ; and this idea he carried so far as to ignore entirely the doctrine of definite proportions. The influence which he ascribed to cohesion and volatility, have been received with more favor by chemists ; yet the rules deduced from his principles are not free from exceptions ; and, as he has given no means of estimating the efficiency of the supposed cause, his explanations seem only to place the doctrine of chemical affinity beyond the pale of exact scientific inquiry. Accordingly, it is on practical operations that we are mainly dependent for our knowledge on this important subject ; and the ancient mariners were not more reluctant to venture out of sight of the land, than modern chemists are to trust to conclusions beyond what is confirmed by direct experiment.

There seems, however, some hopes that the difficulties of definitely estimating the intensity of chemical action are likely to be removed by the aid derived from another department of physical science. The doctrine of the "correlation of forces" has already enlisted in its behalf many zealous advocates, who have done much to support it by experiment, and to show a conformity to its principles in the operations of nature. In scientific circles the doctrine seems now to be generally received, so far as it applies to heat, electricity and m chanical action. Recently much experimental research has been expended, with a view of ascertaining whether the common relations between force extend to chemical affinity, in its varied operations between the different elements and their compounds. To discover some definite measure of the forces concerned in chemical union is the first step to an accurate investigation of the subject. Though the terms "strong and weak" acids and bases have long been in vogue among chemists, there has been hitherto no means by which the chemical energy of each acid and base could be determined with such precision as science requires. From the doctrine of the correlation of forces, it has been concluded that the affinity between the several acids and bases may be accurately measured by the heat evolved during their combination ; that in the separation of elements and of binary compounds from one another, there is expended the same amount of heat as is produced by their union ; and that the course of chemical action is always in favor of those combinations which develop the greatest amount of calorific energy.

The last inference, when tested by experiment, is found to be generally correct, but not universally so. In most cases in solution, a salt is decomposed by an acid which exceeds the one already in combination in the power of producing heat while combining with the base. But to this general rule there are some exceptions which have served to embarrass the investigation ; and Thompson of Copenhagen, though once partial to the calorific theory and long engaged in testing it by experiment, has lately pronounced it inadequate, and he contends that chemical action is controlled by some force independent of heat.

But before embracing the conclusion, it may be advisable to estimate with more precision than has hitherto been aimed at, the effects of certain agencies which are known to influence chemical action. One of these comes into play when the salts arising from decomposition have little or no solubility. When sulphate of soda decomposes acetate of lime in solution, the effect depends, in a great measure, on the precipitation of the sulphate of lime, which is but slightly soluble in water. But instead of supposing with Berthollet, that the devellent affinities in this case are aided by the force of cohesion between the particles of the precipitated salt, it would be more reasonable to suppose that the aid is derived from the latent heat which bodies require for fluidity, and which they evolve on becoming solid.

There is also another cause to which some importance must be assigned. In an article published 24 years ago, I maintained that solution weakens affinity, that as it is dilute the effect is greater, but that it is not felt by insoluble compounds. To this cause I ascribed the decomposition which certain salts of bismuth, antimony and other metals undergo when diluted with a large quantity of water. On this same principle also, I accounted for the fact, that carbonate of potash will not be decomposed by quicklime, unless it be dissolved in about ten times its weight of water. Somewhat similar views as to the effects of water has been recently advanced by Berthollet, and he has endeavored to test them by measuring the heat produced by the mutual action of acids and bases in solutions of different degrees of strength. In many cases he finds that increased dilution has no material result; in other cases, it has a very decided influence on the amount of heat produced, and the supposed cause, though not universal in its operation, has its effects inscribed on many of the phenomena connected with chemical action.

[TO BE CONTINUED.]

